# Identification of Structural Features of Condensed Tannins That Affect Protein Aggregation

**DOI:** 10.1371/journal.pone.0170768

**Published:** 2017-01-26

**Authors:** Honorata M. Ropiak, Peter Lachmann, Aina Ramsay, Rebecca J. Green, Irene Mueller-Harvey

**Affiliations:** 1 School of Agriculture, Policy and Development; Chemistry and Biochemistry Laboratory, University of Reading, Reading, Berkshire, United Kingdom; 2 Independent Researcher, Bookham, Surrey, United Kingdom; 3 School of Chemistry, Food and Pharmacy; University of Reading, Reading, Berkshire, United Kingdom; Islamic Azad University Mashhad Branch, ISLAMIC REPUBLIC OF IRAN

## Abstract

A diverse panel of condensed tannins was used to resolve the confounding effects of size and subunit composition seen previously in tannin-protein interactions. Turbidimetry revealed that size in terms of mean degree of polymerisation (mDP) or average molecular weight (amw) was the most important tannin parameter. The smallest tannin with the relatively largest effect on protein aggregation had an mDP of ~7. The average size was significantly correlated with aggregation of bovine serum albumin, BSA (mDP: *r* = -0.916; amw: *r* = -0.925; *p*<0.01; df = 27), and gelatin (mDP: *r* = -0.961; amw: *r* = -0.981; *p*<0.01; df = 12). The procyanidin/prodelphinidin and *cis*-*/trans*-flavan-3-ol ratios gave no significant correlations. Tryptophan fluorescence quenching indicated that procyanidins and *cis*-flavan-3-ol units contributed most to the tannin interactions on the BSA surface and in the hydrophobic binding pocket (*r* = 0.677; *p*<0.05; df = 9 and *r* = 0.887; *p*<0.01; df = 9, respectively). Circular dichroism revealed that higher proportions of prodelphinidins decreased the apparent α-helix content (*r* = -0.941; *p*<0.01; df = 5) and increased the apparent β-sheet content (*r* = 0.916; *p*<0.05; df = 5) of BSA.

## Introduction

**Fig 1:**
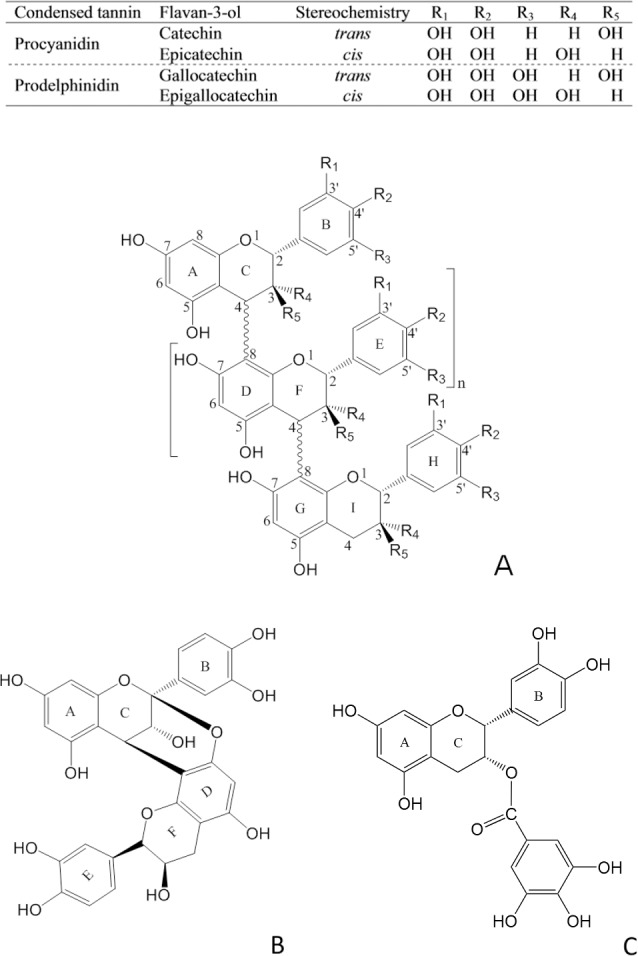
Examples of condensed tannins and a galloylated flavan-3-ol. (A) a B-type condensed tannin; (B) an A-type condensed tannin, epicatechin(4ß→8, 2ß→*O*→7)-epicatechin; and (C) a flavan-3-ol monomer, epicatechin gallate.

Condensed tannins (CT, syn. proanthocyanidins, Fig 1) occur as polyphenolic oligomers and polymers in many fruits and in some vegetables [1], medicinal plants [2] and forage legumes [3]. Some CT can have positive impacts on animal nutrition, health and welfare [3], and there is now also considerable interest in their anthelmintic effects against gastrointestinal nematodes [4–9]. Recent research has shown that the integrity of the parasitic nematode cuticle becomes distorted after exposure to CT [10]. This cuticle is largely composed of collagen-like proline-rich proteins and structural proteins (cuticlins) [11, 12]. CT have a high affinity to proline-rich proteins [13], which may explain the effect of CT on nematode cuticles.

Several different techniques exist for studying CT-protein interactions [14, 15] and each probes different aspects. The initial molecular interactions can be assessed for example by nuclear magnetic resonance spectroscopy, electrospray ionisation mass spectrometry (ESI-MS) or isothermal titration calorimetry (ITC) for hydrogen bonding, hydrophobic interactions or for aromatic stacking [14, 15]; and as CT crystal structures are not yet available [16] molecular modelling has also been employed to explore CT-protein interactions [17]. Subsequent cross-linking of these initial CT-protein complexes and their aggregation can be studied by nephelometry, dynamic light scattering and turbidimetry; and precipitation by protein precipitation methods. In addition, the following parameters can be obtained: stoichiometry of binding by ITC and MS [15], changes to protein structure by circular dichroism (CD) [17], and binding affinity and accessibility to fluorophores such as tryptophan by fluorescence quenching [18].

Despite a large number of previous studies on CT-protein interactions, it is still not clear, which particular CT features contribute most to complex formation. One reason for this is the presence of confounding effects within CT mixtures that can be found in plants. For example, CT fractions isolated from sainfoin, a forage legume, yielded a positive correlation between the mean degree of polymerisation (mDP) and the molar percentage of prodelphinidins (PD), which prevented identification of the key factor(s) responsible for saturating the available binding sites of bovine serum albumin (BSA) and gelatin [19]. According to another study, mDP affected BSA and alfalfa leaf protein precipitation; however, this work used only PD-rich fractions from white clover flowers and big trefoil leaves [20]. Other studies used a series or mixtures of oligomeric procyanidins (PC) from cocoa beans composed of epicatechin units only and showed that size was an important factor in CT-BSA precipitation [21] and binding [22]. It has similarly been reported that binding to elastase increased with the size of PC oligomers from grape seeds [17]. However, nephelometric studies on BSA, α-amylase and proline-rich proteins showed that aggregation increased not only with increasing degree of polymerisation [23] but also with galloylation of PC [13]. Some reports indicated that flavan-3-ol monomers with galloyl groups [13, 24] or *trans* stereochemistry had higher affinity towards proline-rich proteins [13]. In contrast, other studies could not correlate CT features with protein precipitation [25, 26].

Therefore, the aim of this work was to establish, which CT parameters contributed most to aggregation upon binding to proteins. A large panel of CT was isolated in order to feature a wide range of average sizes, PC/PD ratios and *cis*-*/trans*-flavan-3-ol ratios. Two model proteins were used for the interaction studies; i.e. BSA, which is a relatively rigid globular protein; and gelatin, which is a highly flexible proline-rich protein. Turbidimetry is particularly suited for screening a large number of CT samples and can thus be used to investigate structure-activity relationships [15]. In turbidimetry, the reduction in light transmission is measured, when a stable, cloudy haze is formed [15] at the time of reaction. Here, we used turbidimetry in combination with curve fitting to systematically explore the effect of a large number of CT samples on protein aggregation. In addition, complementary studies used fluorescence quenching and CD spectroscopy to assess the CT-BSA interactions. These techniques are commonly used to study ligand-protein interactions [27–29].

## Materials and Methods

### Materials

Sephadex LH-20 was obtained from GE Healthcare (Little Chalfont, UK); acetone (analytical reagent grade), acetonitrile (HPLC grade), dichloromethane (laboratory reagent grade), hexane (GLC, pesticide residue grade) and methanol (HPLC grade) were from ThermoFisher Scientific (Loughborough, UK). Bovine serum albumin (BSA, heat shock fraction, protease free, fatty acid free, essentially globulin free, ≥98%, 66 kDa), gelatin (from bovine skin, Type B, BioReagent, suitable for cell culture, ~225 g Bloom), citric acid monohydrate, trisodium citrate dehydrate, BIS-TRIS (≥98%), Tricine (≥98%), sodium phosphate monobasic dihydrate and polyvinylpolypyrrolidone (PVPP, ~110 μm particle size) were purchased from Sigma-Aldrich (Poole, UK) and disodium hydrogen phosphate dihydrate from Fluka (Sigma-Aldrich, Poole, UK). Deionised water was purified in an Option 3 water purifier (ELGA Process Water, Marlow, UK) and ultrapure water (MQ H_2_O) in a Milli-Q Plus system (Millipore, Watford, UK).

### Plant Materials

Samples of medicinal plants and herbal products were obtained and prepared as described before [2]: blackthorn flowers (*Pruni spinosae flos*), hawthorn flowers (*Crataegi inflorescentia*), heather flowers (*Callunae vulgaris flos*), hop strobile (*Lupuli flos*), *Tilia* flowers (*Tiliae inflorescentia*), pine buds (*Pini gemmae*), bilberry leaves (*Myrtilli folium*), birch leaves (*Betulae folium*), blackcurrant leaves (no. 1) (*Ribis nigri folium*), cowberry leaves (*Vitis idaeae folium*), great water dock roots (*Hydrolapathi radix*) and willow bark (*Salicis cortex*) were from Flos (Mokrsko, Poland); walnut leaves (*Juglandis folium*) were from Kawon (Gostyń, Poland); and white clover (*Trifolium repens*) flowers were from Zioła z Kurpi (Jednorożec, Poland) [2]. Flowering aerial parts of sainfoin (*Onobrychis viciifolia*, var. Esparsette) were provided by Peter Davy (Barham, UK), hazelnut (*Corylus avellana*) pericarps were supplied by Société Inovfruit (Musidan, France), blackcurrant (*Ribes nigrum*) leaves (no. 2) and redcurrant (*Ribes rubrum*) leaves were collected from Hildred’s Pick-Your-Own Farm (Goring-upon-Thames, UK) [7]. Yellow iris (*Iris pseudacorus*) leaves were collected in Bookham (Surrey, UK), cider apple beverage (‘Three Countries, premium strong dry cider’, 5.5% alcohol) was obtained from Aston Manor Brewery Co Ltd (Aston, UK) and dried cider apple powder (‘the original prestige cider kit’) was obtained from Gert Strand AB (Sweden). Sainfoin was wilted overnight, freeze-dried and ground [2] to successively pass 8, 5 and 1 mm sieves; hazelnut pericarps were ground to pass a 1 mm sieve; redcurrant and blackcurrant (no. 2) leaves were air-dried and then ground to pass 5 and 1 mm sieves, yellow iris leaves were freeze-dried and ground to pass 5 and 1 mm sieves. All plant materials were stored at room temperature in the dark. CT from other plant materials were extracted, fractionated, analysed and characterised as already described: shea (*Vitellaria paradoxa*) meal was provided by AarhusKarlshamn Sweden AB (Sweden) [4], cinnamon (*Cinnamomum verum*) bark was obtained from Dary Natury (Grodzisk, Poland) [9], cocoa (*Theobroma cacao*) beans were obtained from Detox Your World (RawCreation Ltd, Norfolk, UK) [7] and lespedeza (*Lespedeza cuneata*) pellet (leaf meal) was from Sims Brothers Seed Company (Union Springs, AL, USA) [30].

### CT Extraction

Hazelnut pericarps, pine buds and walnut leaves were de-fatted with hexane prior to extraction [7]. Acetone/water (70% aqueous acetone, 250–500 mL) was used to prepare extracts from plant materials (20–50 g) [2]. After evaporation of acetone, the aqueous extracts were frozen overnight at -20°C.

### CT Fractionation

Aqueous extracts were fractionated on Sephadex LH-20 by gravity flow [7]. In brief, on the day of fractionation the aqueous extracts were thawed, centrifuged for 3 min at 4500 rpm (Jouan CR3i Multifunction Centrifuge, Thermo Electron Corporation, Basingstoke, UK) to remove insoluble particles. The aqueous extract was applied to the resin, followed by a rinse with H_2_O to remove sugars, flavanol monomers and other contaminants. CT fractions were then eluted using acetone/water (30, 50 and 80% aqueous acetone) to obtain three CT fractions: fraction 1 (F1), fraction 2 (F2) and fraction 3 (F3), respectively. Acetone was removed on a rotary evaporator with a water bath at 35°C and the remaining aqueous fractions were freeze-dried. Hawthorn and blackthorn flowers were fractionated as described before [9].

The commercial cider (12 L) was degassed by stirring with a magnetic stirrer for 1 h, followed by ethanol evaporation and sample concentration on a rotary evaporator and partially freeze-dried due to the high sugar content. The sample was diluted in 10 L H_2_O prior to loading on the Sephadex LH-20 resin, fractionated, freeze-dried and the fractionation was repeated.

### CT Analysis

The CT fractions were derivatised with benzyl mercaptan, the thiolysis reaction products were identified by LC-MS and quantified by RP-HPLC-DAD [2]. Due to low yields, F3 fractions were analysed by RP-HPLC-DAD only. The great water dock root F1 and hazelnut pericarp F1 were also assayed in triplicates for free flavan-3-ols [2]. No free flavan-3-ols were detected in F2 samples.

### Calculation of CT Parameters

The mDP-values, PC/PD ratio and *cis*-/*trans*-flavan-3-ol ratio, molar percentages of A-type linkages and galloylation and relative molar percentages of flavan-3-ol subunits were calculated as described [2]. Average molecular weight (amw) was calculated [31] for all samples using the following equation (% refers to molar percentages of flavan-3-ol subunits):

$$amw=\left\{mDP\times [\left(\frac{\%PC}{100}\times 290.26)+(\frac{\%PD}{100}\times 306.27)+(\frac{\%galloylation}{100}\times 152.12\right)]\right\}-(mDP\times 2-2)-\left(\frac{\%A-type}{100}\times 2\right)-\left(\frac{\%galloylation}{100}\times 1\right)$$

### Turbidimetry: CT-BSA

Measurements were performed in citrate buffer (50 mM, pH 6) as described before [32] with the following changes. Each CT fraction (200 L, 3 mg/mL for F2 samples and 10 mg/mL for F1 samples) was titrated as a sequence of 5 μL aliquots (10 μL for cocoa bean F2 and blackthorn flower F2) into a BSA solution (2 mL, 5 μM) within 15 min at room temperature. The measurement interval was 20 s. At the end of the titration the formation of a stable hazy solution was observed. Typically, CT were studied with three replicate titrations. Absorbance readings were acquired in triplicate with 3 s intervals at 400 nm using a JASCO V-530 spectrophotometer (JASCO UK Ltd, Essex, UK) in a 1 cm polystyrene cuvette and buffer was used as the blank.

Absorbances were averaged and converted to % transmission (%T). The concentration (μM or mg/mL) of ligands was corrected for dilution and a graph was created of %T versus [CT]/[protein]. The titration data from each replicate were averaged and fitted to a single sigmoid function using Pro-Data™ Software Suite version 4.4.2.0 (Applied Photophysics Ltd, Leatherhead, UK). The CT/protein concentration ratio, expressed either on a molar (M/M) or a mass basis [(mg/mL)/(mg/mL)], at half maximum (i.e. 50% transmission) of the function was used as an indicator of the efficacy of CT on protein aggregation, henceforward referred to as half maximal effective ratio (ER_50_). All reported turbidimetry data were corrected for protein dilution and CT content (g CT/100 g of fraction) as measured by thiolysis.

### Turbidimetry: CT-Gelatin

Gelatin was solubilised by heating to 40°C in 50 mM BIS-TRIS/HCl buffer at pH 7. Measurements were performed as above by titrating a CT fraction (400 μL, 3 mg/mL) in a sequence of 10 μL aliquots into a gelatin solution (2 mL, 1 mg/mL, i.e. ~20 μM [33]) in duplicates. The data were fitted to a single exponential function using Microsoft Excel to calculate ER_50_ values.

### Turbidimetry: pH Effect

The influence of pH on the efficacy of CT-protein aggregation was measured as outlined above by titration of the sainfoin F2 sample into BSA with the following buffers at 50 mM: pH 3, 4, 5 and 6 –citrate buffer; pH 6 and 7 –BIS-TRIS/HCl and pH 8 –Tricine/KOH. Typically, triplicate points of titration were averaged and data were fitted to a single sigmoid function.

### Circular Dichroism

A CT fraction (15 μL, 3 mg/mL in 5 mM sodium phosphate buffer at pH 6) was added to BSA (0.4 mL, 2.5 μM in 5 mM sodium phosphate buffer at pH 6) and measured within 30 s. There was no visible aggregation present before and after the measurement. CD spectra were recorded at 180–300 nm with a 1 nm bandwidth on a Chirascan™-Plus CD Spectrometer with the Pro-Data™ Software Suite (both Applied Photophysics Ltd, Leatherhead, UK) in a 0.1 cm path length quartz cuvette (birefringence minimised) with acquisition of 3 CD spectra at room temperature. The contribution of CT fractions to the BSA CD spectra was removed by subtraction of the spectrum of the CT fraction alone in buffer. The CD difference spectra were calculated by subtracting the ‘CT only’ CD spectra (CD_CT_) and the ‘BSA only’ CD spectra (CD_BSA_) from the spectrum recorded with the CT and BSA mix (CD_BSA_CT_): ΔCD = CD_BSA_CT_ - (CD_CT_ + CD_BSA_). Differences in CD spectra were quantified using qBiC Biocomparability Suite version 1.0.1 (Applied Photophysics Ltd, Leatherhead, UK). An averaged CD spectrum of ligand-free BSA was set as a reference. CD spectra were normalised to absolute area and weighted spectral differences were calculated. Properties of the secondary structure were calculated with DichroWeb [34, 35] using the Contin-LL method (Provencher & Glockner Method) with a reference dataset of SMP180 (optimised for 180–240 nm) [36] and the ‘closest matching solution with all proteins’ was chosen. The CDSSTR method gave similar results (data not shown).

### Fluorescence Quenching Measurements

Measurements were performed using a dilution series of the CT fractions in 50 mM citrate buffer at pH 6. Seven additions of 10 μL at 0 mg CT/mL into BSA (2 mL, 5 μM) were used for the initial equilibration, followed by 10 additions of 5 μL of each of the following concentrations 0.09, 0.19, 0.38, 0.75, 1.5 and 3 mg CT/mL. Each titration was carried out within 40 min with a 30 s delay between each addition and measurement. The fluorescence intensities were recorded on a Varian Cary Eclipse fluorescence spectrophotometer with a Cary temperature controller and stirrer control (Agilent Technologies Ltd, UK) in a 1 cm path length quartz cuvette with a micro magnetic stirrer at 25°C. The excitation wavelength was 295 nm, excitation and emission bandwidths were 5 nm, and the emission spectrum was recorded between 300 and 500 nm. Only those CT fractions that did not exhibit any fluorescence at concentrations of ~0.14 and ~0.27 mg/mL in buffer were used. Typically, CT were studied with three replicate titrations (with 60 data points each). The intensities recorded at 340 nm were used for the generation of Stern-Volmer plots. The Stern-Volmer quenching constant (*K*_SV_) was calculated from a linear regression [37] in the initial linear part of the graph. That graph was composed of a ratio of fluorescence intensities, before (*F*_0_) and after addition of quencher (*F*), versus quencher concentration (CT in this study), i.e. *F*_0_/*F* versus [CT]. Data were calculated using the Stern-Volmer equation [37]:

$$\frac{F_{0}}{F}=1+k_{q}\tau_{0}[Q]=1+K_{sv}[Q]$$

where: *F*_0_ –fluorescence intensity in the absence of quencher; *F–*fluorescence intensity in the presence of quencher; *k*_q_−bimolecular quenching constant; τ_0_ –the lifetime of the fluorophore in the absence of quencher; *Q–*quencher; *K*_SV_−Stern-Volmer constant. All data from the fluorescence studies were corrected for BSA dilution and CT content (g CT/100 g fraction).

### Statistical Analyses

In general, Dancey and Reidy’s categorisation [38] was used to indicate the degree of correlation; i.e. perfect, strong, moderate, weak or zero; of the data in the plots. The Shapiro-Wilk test of normality was used to test for normally distributed data (*p*>0.05). To test for statistical significance Pearson’s correlation coefficient was used for normally distributed data (*r*, 2-tailed test; *p*<0.05 or 0.01, where indicated) and Spearman’s rho was used for not normally distributed data (*r*_s_, 2-tailed test; *p*<0.05 or 0.01, where indicated). Only statistically significant evaluations are noted in figure captions and throughout the text. Shapiro-Wilk test, Pearson’s correlation and Spearman’s rho were performed using the IBM^®^ SPSS^®^ Statistics version 21 software.

Turbidimetry study: all variables (CT characteristics and ER_50_ values) were not normally distributed. Variables were transformed to normality with the natural logarithm, although PC (%) and *cis-*flavan-3-ols (%) remained not normally distributed. Therefore, Pearson’s correlation and Spearman’s rho were used accordingly.

CD study: variables without natural logarithm transformation (α-helix and β-sheet content, molar percentages of PC and mDP-values) were subjected to Pearson’s correlation.

Fluorescence study: variables without natural logarithm transformation (mDP-values, amw, molar percentages of PC and ER_50_ values) were subjected to Pearson’s correlation. Then all variables (including not normally distributed molar percentages of *cis*-flavan-3-ols) were subjected to Spearman’s rho.

## Results and Discussion

### CT Composition

To probe CT-protein aggregation, we isolated and characterised CT fractions from 23 different plant materials [2, 4–9, 30, 39]. Most of these fractions had CT with B-type linkages and a wide range of PC/PD and *cis/trans*-flavan-3-ol ratios; six fractions also had CT with galloylated flavan-3-ol subunits and another six fractions had CT with A-type linkages (Fig 1, Table 1 and S1 Table). The average molecular weights of these CT varied from 1028 to 7580 Da, PC/PD ratios from 100:0 to 1:99, *cis*-*/trans*-flavan-3-ol ratios from 99:1 to 12:88, the molar percentages of galloylation from 0 to 54% and A-type bonds from 0 to 21% (Table 1). Among these 35 samples, there was no obvious correlation among CT characteristics (S1 Fig) despite statistically moderate correlations between PD and mDP, and between PD and *cis*-flavan-3-ols (S1A and S1C Fig). However, this moderate correlation between mDP and PD was not present (*p*>0.05) for CT fractions that were chosen for the CT-gelatin aggregation and corresponding CT-BSA aggregation by turbidimetry or tryptophan fluorescence quenching. Therefore, this diverse CT panel was suitable for testing the mDP and PD effects separately.

**Table 1:** Condensed tannin (CT) contents, mean degrees of polymerisation (mDP), average molecular weights (amw), procyanidin/prodelphinidin (PC/PD) and *cis*-/*trans*-flavan-3-ol ratios, molar percentages of galloylation and A-type linkages in fractions isolated from various plant materials. *Note*: Results from a few of these fractions were reported previously [4–9, 39] and are included here for clarity purposes.

CT fraction	CT (g/100 g)	mDP	amw (Da) ^b^	PC ∕ PD			*cis* ∕ *trans*			% galloylation	ref.
Great water dock root F1 ^a^	31.9 (±0.6)	3.0 (±0.1)	1028	100.0	∕	0.0 (±0.1)	85.3	∕	14.7 (±0.1)	34.6 (±0.1)	
Great water dock root F2	63.7 (±1.7)	5.1 (±0.1)	1906	88.6	∕	11.4 (±0.1)	95.6	∕	4.4 (±0.1)	54.3 (±0.1)	[39]
Great water dock root F3	48.9 (±2.1)	14.4 (±0.0)	4989	66.9	∕	33.1 (±0.1)	94.7	∕	5.3 (±0.0)	34.0 (±0.0)	
Hazelnut pericarp F1 ^a^	64.5 (±2.3)	4.7 (±0.0)	1366	91.2	∕	8.8 (±0.0)	39.8	∕	60.2 (±0.1)	1.6 (±0.0)	
Hazelnut pericarp F2	68.6 (±5.0)	10.5 (±0.1)	3102	88.0	∕	12.0 (±0.1)	54.2	∕	45.8 (±0.1)	3.7 (±0.1)	
Shea meal F2	44.9 (±0.8)	4.1 (±0.1)	1519	27.5	∕	72.5 (±0.1)	59.8	∕	40.2 (±0.1)	46.1 (±0.2)	[4, 5]
										**% A-type bond**	
Bilberry leave F2	63.2 (±2.7)	9.5 (±0.0)	2732	97.3	∕	2.7 (±0.0)	96.6	∕	3.4 (±0.0)	5.7 (±0.0)	
Blackthorn flower F2	33.0 (±5.8)	4.1 (±0.1)	1176	100.0	∕	0.0 (±0.1)	72.8	∕	27.2 (±0.1)	21.3 (±0.1)	
Cinnamon bark F2	55.0 (±0.9)	7.0 (±0.1)	2019	100.0	∕	0.0 (±0.0)	85.9	∕	14.1 (±0.1)	18.0 (±0.1)	[9]
Cowberry leaf F2	68.1 (±2.7)	8.7 (±0.0)	2495	100.0	∕	0.0 (±0.0)	72.1	∕	27.9 (±0.0)	17.2 (±0.0)	
Heather flower F2	67.2 (±1.7)	8.2 (±0.0)	2372	89.3	∕	10.7 (±0.0)	88.0	∕	12.0 (±0.0)	7.9 (±0.0)	
Heather flowerF3	54.6 (±1.3)	25.5 (±0.0)	7403	88.1	∕	11.9 (±0.0)	95.1	∕	4.9 (±0.0)	1.3 (±0.0)	
Birch leaf F2	63.6 (±2.5)	8.3 (±0.1)	2464	41.1	∕	58.9 (±0.1)	70.7	∕	29.3 (±0.1)	^nd^	[5, 8, 39]
Birch leaf F3	53.2 (±1.8)	17.7 (±0.1)	5289	37.8	∕	62.2 (±0.0)	80.3	∕	19.7 (±0.0)	^nd^	
Blackcurrant leaf (no. 1) F2	77.1 (±3.9)	11.8 (±0.1)	3591	4.7	∕	95.3 (±0.0)	18.8	∕	81.2 (±0.1)	^nd^	[5, 6, 8, 39]
Blackcurrant leaf (no. 2) F2	86.6 (±2.7)	7.8 (±0.2)	2364	5.1	∕	94.9 (±0.0)	12.0	∕	88.0 (±0.1)	^nd^	[39]
Blackcurrant leaf (no. 2) F3	69.9 (±0.9)	16.6 (±0.1)	5025	5.6	∕	94.4 (±0.1)	19.8	∕	80.2 (±0.1)	^nd^	[39]
Cider apple beverage F2	35.9 (±2.7)	7.5 (±0.1)	2218	61.0	∕	39.0 (±0.1)	87.6	∕	12.4 (±0.1)	^nd^	
Cider apple powder F2	37.4 (±1.1)	6.8 (±0.0)	1954	100.0	∕	0.0 (±0.0)	96.3	∕	3.7 (±0.0)	^nd^	
Cocoa bean F2	75.5 (±2.9)	5.4 (±0.1)	1567	100.0	∕	0.0 (±0.0)	96.3	∕	3.7 (±0.1)	^nd^	[5, 7, 39]
Hawthorn flower F2	48.8 (±1.6)	10.7 (±0.0)	3087	100.0	∕	0.0 (±0.0)	98.5	∕	1.5 (±0.0)	^nd^	
Hop strobile F2	66.6 (±1.7)	10.6 (±0.0)	3106	75.0	∕	25.0 (±0.0)	75.5	∕	24.5 (±0.2)	^nd^	
Lespedeza pellet F3	69.7 (±1.7)	25.0 (±0.3)	7580	5.6	∕	94.4 (±0.0)	80.8	∕	19.2 (±0.1)	^nd^	
Pine bud F2	93.7 (±2.5)	10.5 (±0.1)	3146	36.2	∕	63.8 (±0.1)	70.7	∕	29.3 (±0.1)	^nd^	
Pine bud F3	72.7 (±4.9)	17.6 (±0.1)	5240	44.2	∕	55.8 (±0.1)	83.8	∕	16.2 (±0.1)	^nd^	
Redcurrant leaf F2	91.5 (±4.2)	11.0 (±0.1)	3350	7.5	∕	92.5 (±0.0)	65.3	∕	34.7 (±0.1)	^nd^	[39]
Sainfoin aerial part F2	82.6 (±2.0)	12.5 (±0.1)	3733	31.7	∕	68.3 (±0.1)	82.6	∕	17.4 (±0.1)	^nd^	[39]
*Tilia* flower F2	91.7 (±3.8)	7.9 (±0.1)	2270	99.1	∕	0.9 (±0.1)	95.6	∕	4.4 (±0.1)	^nd^	[5, 6, 8, 39]
*Tilia* flower F3	58.1 (±7.0)	20.9 (±0.2)	6043	98.6	∕	1.4 (±0.2)	99.0	∕	1.0 (±0.2)	^nd^	
Walnut leaf F2	69.0 (±1.7)	12.3 (±0.1)	3617	69.1	∕	30.9 (±0.0)	76.3	∕	23.7 (±0.0)	^nd^	[5, 8]
Walnut leaf F3	64.6 (±6.5)	18.8 (±0.1)	5594	43.4	∕	56.6 (±0.1)	76.8	∕	23.2 (±0.1)	^nd^	
White clover flower F2	82.4 (±2.0)	12.7 (±0.0)	3875	1.2	∕	98.8 (±0.0)	61.8	∕	38.2 (±0.0)	^nd^	[5, 6, 8, 39]
Willow bark F2	83.3 (±0.6)	9.9 (±0.0)	2865	94.0	∕	6.0 (±0.0)	78.1	∕	21.9 (±0.0)	^nd^	[5, 8]
Willow bark F3	67.4 (±0.7)	15.0 (±0.1)	4352	95.1	∕	4.9 (±0.1)	83.9	∕	16.1 (±0.1)	^nd^	
Yellow iris leaf F2	85.1 (±2.8)	9.2 (±0.1)	2703	69.8	∕	30.2 (±0.1)	63.3	∕	36.7 (±0.1)	^nd^	[39]

^a^ data were corrected for free flavan-3-ols.

^b^ data were not reported previously; ^nd^ not detected; CT content has been reported [6] (calculated with mass response factor); standard deviation in parentheses.

### Effect of pH on CT-BSA Aggregation by Turbidimetry

**Fig 2:**
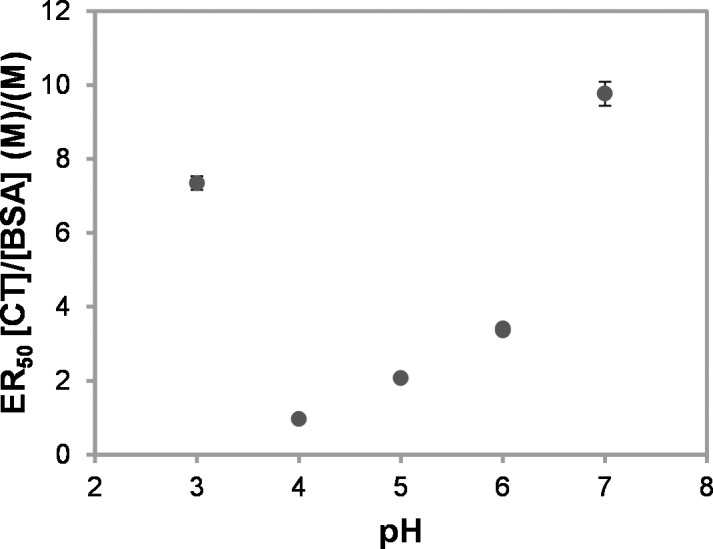
The effect of pH on condensed tannin (CT) efficacy to aggregate BSA. ER_50_ is the half maximal effective ratio (values were corrected for CT content): 7.3 (±0.2) at pH 3, 1.0 (±0.0) at pH 4, 2.1 (±0.0) at pH 5, 3.4 (±0.0) at pH 6 (for citrate and BIS-TRIS buffer), 9.8 (±0.3) at pH 7, and no aggregation was observed at pH 8. The values in parentheses and error bars indicate the estimated error of the fit of the titration data for ER_50_ (after averaging experimental data points, typically n = 3 replicates).

There are many factors that can impact on CT-protein interactions and pH is one of them [14]. Therefore, we performed an initial evaluation of the effect of pH on the efficacy of sainfoin aerial part F2 CT to precipitate BSA by turbidimetry between pH 3 to 8. The results were as follows: ER_50_ was 7.3 at pH 3, 1.0 at pH 4, 2.1 at pH 5, 3.4 at pH 6 (for 2 different buffers) and 9.8 for pH 7 (Fig 2). ER_50_ at pH 8 was not determined as aggregation was not observed. As expected, the lowest ER_50_ values were measured at pH 4 and 5, which are close to the pI of BSA, i.e. is 5.3 for fatty acid depleted BSA [40] and agreed with the literature, as maximum precipitation tends to occur at pH values close to the pI [41]. Therefore, these experiments showed the same trend and validated a different technique. They also demonstrated that turbidimetry could be applied over a wide range of pH values and even outside the pI of the protein.

### CT-BSA Aggregation by Turbidimetry

**Fig 3:**
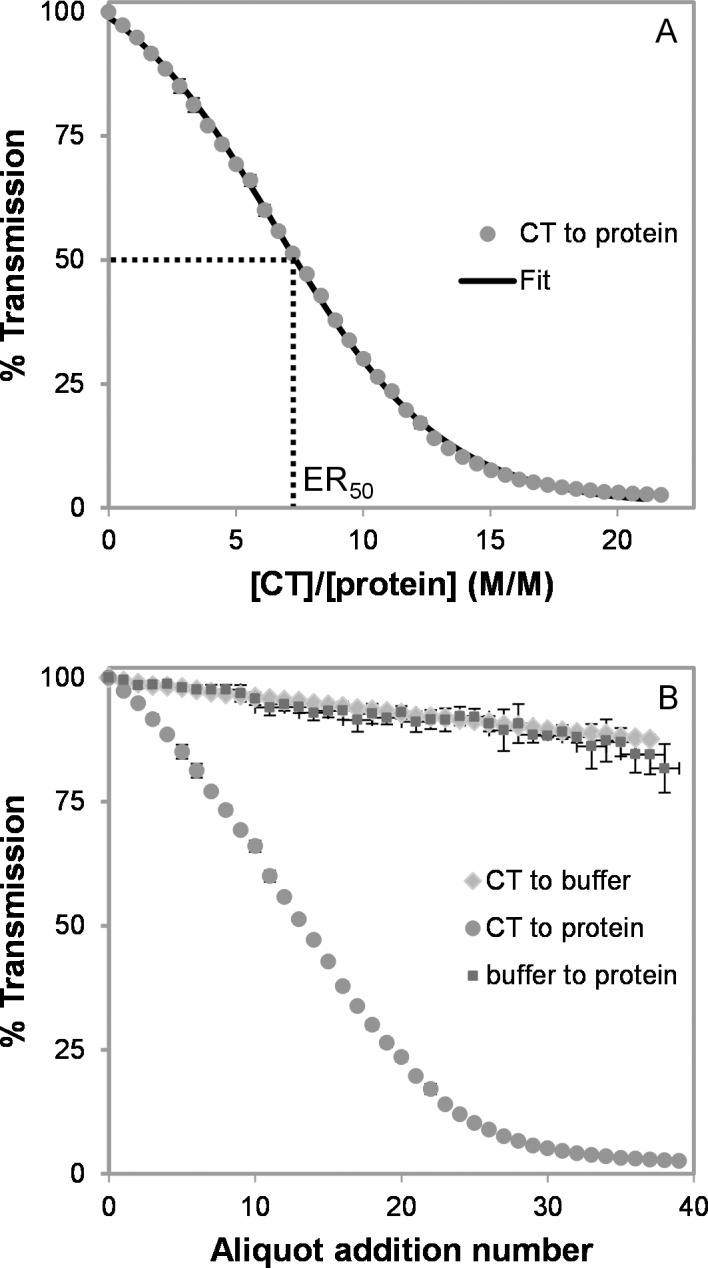
Experimental turbidimetry data obtained by adding condensed tannins (CT, *Tilia* flower F2 sample) to BSA. (A) estimation of half maximal effective ratio (ER_50_) by a single sigmoid fit; (B) controls (CT addition to buffer/BSA, buffer addition to BSA).

For each CT-protein mixture, turbidity was measured at increasing CT/protein ratios. The example in Fig 3 shows the change in turbidity during a typical CT-protein titration experiment. Turbidimetry data were plotted as %T versus the concentration ratio of ligands, i.e. [CT]/[protein], in order to calculate the efficacy (ER_50_) of different CT types to aggregate the protein (Fig 3A). This approach was used here to transform the qualitative turbidimetry results [15] into a quantitative value to enable comparison of the different CT samples.

**Fig 4:**
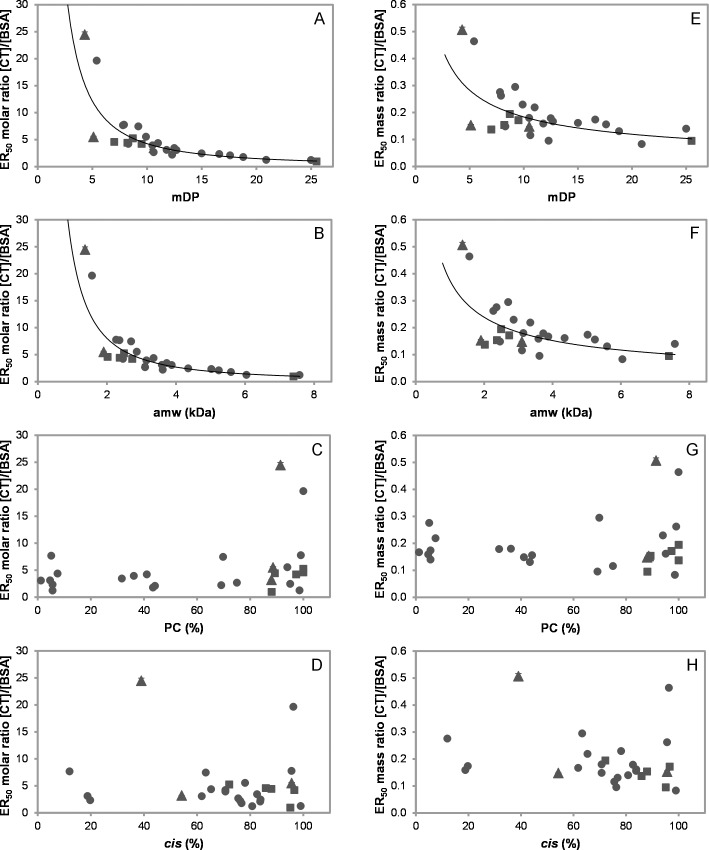
Influence of condensed tannin (CT) characteristics on aggregation of BSA. ER_50_ –half maximal effective ratio; mDP–mean degree of polymerisation; amw–average molecular weight of CT; PC–procyanidins; *cis*–*cis*-flavan-3-ols.,● –B-type CT, ▲ –B-type galloylated CT, ■ –B-type with A-type linkages. Values corrected for CT content; CT fractions of >50 g CT/100 g of fraction; error bars are depicted, for more detail see Table 2; (A, B, E, F) fitted to power function; (A) ER_50_ [CT]/[BSA] (M/M) versus mDP, *R*^2^ = 0.84 (*r* = -0.916; *p*<0.01; df = 27; and *r*_s_ = -0.926; *p*<0.01; df = 27); (B) ER_50_ [CT]/[BSA] (M/M) versus amw (kDa), *R*^2^ = 0.86 (*r* = -0.925; *p*<0.01; df = 27; and *r*_s_ = -0.925; *p*<0.01; df = 27); (C) ER_50_ [CT]/[BSA] (M/M) versus PC (%); (D) ER_50_ [CT]/[BSA] (M/M) versus *cis* (%); (E) ER_50_ [CT]/[BSA] (mg/mL)/(mg/mL) versus mDP, *R*^2^ = 0.44 (*r* = -0.664; *p*<0.01; df = 27; and *r*_s_ = -0.526; *p*<0.01; df = 27); (F) ER_50_ [CT]/[BSA] (mg/mL)/(mg/mL) versus amw (kDa), *R*^2^ = 0.45 (*r* = -0.674; *p*<0.01; df = 27 and *r*_s_ = -0.521; *p*<0.01; df = 27); (G) ER_50_ [CT]/[BSA] (mg/mL)/(mg/mL) versus PC (%); (H) ER_50_ [CT]/[BSA] (mg/mL)/(mg/mL) versus *cis* (%).

In order to establish, which structural features of CT were most important for protein aggregation, we plotted the ER_50_ values on a molar basis (M/M; Table 2) against the CT parameters (Table 1). All samples with >50 g CT/100 g fraction gave strong significant correlations between ER_50_ versus mDP or amw (Fig 4A and 4B). Fig 4A reveals that the molar ratio of CT:BSA needed to aggregate BSA decreases with increasing mDP. For example, 20 moles of CT from the cocoa bean F2 (mDP = 5.4, 1567 Da) were required to reduce light transmission at 400 nm by 50% compared to just 1 mole of CT from the *Tilia* flower F3 (mDP = 20.9, 6043 Da); Tables 1 and 2. When fractions with lower CT contents were also included, these correlations had a slightly lower magnitude: ER_50_ versus mDP-values (*r* = -0.793; *p*<0.01; df = 33) and ER_50_ versus amw (*r* = -0.813; *p*<0.01; df = 33). Very strong significant correlations were observed if the ER_50_ values of only the B-type CT samples were plotted against mDP (*r* = -0.941; *p*<0.01; df = 19) or amw (*r* = -0.940; *p*<0.01; df = 19), which suggests that 2 to 46% galloylation or 1 to 21% A-type linkages were not important drivers of CT-BSA aggregation.

**Table 2:** Half maximal effective ratio (ER_50_) from condensed tannins (CT)-protein aggregation studies by turbidimetry.

CT fraction	ER_50_			
	[CT]/[BSA] (M/M)	[CT]/[BSA] [(mg/mL)/(mg/mL)]	[CT]/[gelatin] (M/M) ^d^	[CT]/[gelatin] [(mg/mL)/(mg/mL)] ^d^
Great water dock root F1 ^a^^,^ ^c^	5.4 (±0.7)	0.08 (±0.01)	4.3 & 4.0	0.09 & 0.08
Great water dock root F2	5.5 (±0.1)	0.15 (±0.00)	1.7 & 1.6	0.06 & 0.06
Hazelnut pericarp F1 ^a^^,^ ^b^	24.5 (±0.4)	0.51 (±0.01)	-	-
Hazelnut pericarp F2 ^b^	3.2 (±0.0)	0.15 (±0.00)	-	-
Shea meal F2 ^b^^,^ ^c^	5.8 (±0.1)	0.11 (±0.00)	-	-
Bilberry leaf F2 ^b^	4.2 (±0.0)	0.17 (±0.00)	-	-
Blackthorn flower F2 ^b^^,^ ^c^	14.6 (±0.1)	0.26 (±0.00)	-	-
Cinnamon bark F2 ^b^	4.6 (±0.1)	0.14 (±0.00)	-	-
Cowberry leaf F2 ^b^	5.2 (±0.0)	0.19 (±0.00)	-	-
Heather flower F2 ^b^	4.4 (±0.1)	0.15 (±0.00)	-	-
Heather flower F3 ^b^	1.0 (±0.0)	0.09 (±0.00)	-	-
Birch leaf F2	4.2 (±0.1)	0.15 (±0.00)	1.5 & 1.5	0.07 & 0.07
Blackcurrant leaf (no. 1) F2	3.1 (±0.0)	0.16 (±0.00)	0.5 & 0.6	0.04 & 0.04
Blackcurrant leaf (no. 2) F2	7.6 (±0.1)	0.28 (±0.00)	-	-
Blackcurrant leaf (no. 2) F3	2.3 (±0.0)	0.17 (±0.00)	-	-
Cider apple beverage F2 ^c^	1.5 (±0.1)	0.05 (±0.00)	-	-
Cider apple powder F2 ^c^	4.1 (±0.2)	0.09 (±0.00)	-	-
Cocoa bean F2	19.6 (±0.2)	0.46 (±0.00)	3.3 & 3.3	0.10 & 0.10
Hawthorn flower F2 ^c^	2.7 (±0.0)	0.13 (±0.00)	-	-
Hop strobile F2 ^b^	2.7 (±0.1)	0.12 (±0.00)	-	-
Lespedeza pellet F3	1.2 (±0.0)	0.14 (±0.00)	-	-
Pine bud F2	3.9 (±0.1)	0.18 (±0.00)	-	-
Pine bud F3	2.1 (±0.0)	0.16 (±0.00)	-	-
Redcurrant leaf F2	4.4 (±0.1)	0.22 (±0.00)	-	-
Sainfoin aerial part F2	3.4 (±0.0)	0.18 (±0.00)	0.7 & 0.6	0.05 & 0.05
*Tilia* flower F2	7.7 (±0.1)	0.26 (±0.00)	1.3 & 0.9	0.06 & 0.04
*Tilia* flower F3 ^b^	1.2 (±0.0)	0.08 (±0.00)	-	-
Walnut leaf F2 ^b^	2.2 (±0.1)	0.10 (±0.00)	0.5 & 0.5	0.04 & 0.04
Walnut leaf F3 ^b^	1.8 (±0.0)	0.13 (±0.00)	0.3 & 0.3	0.03 & 0.03
White clover flower F2	3.1 (±0.0)	0.17 (±0.00)	0.5 & 0.6	0.04 & 0.04
Willow bark F2 ^b^	5.5 (±0.1)	0.23 (±0.00)	1.0 & 1.1	0.06 & 0.06
Willow bark F3 ^b^	2.4 (±0.0)	0.16 (±0.00)	0.3 & 0.6	0.03 & 0.05
Yellow iris leaf F2	7.4 (±0.1)	0.29 (±0.00)	-	-

^a^ data corrected for free flavan-3-ols.

^b^ fraction in suspension during measurement, assumption was made that all CT were dissolved.

^c^ samples with CT <50 g/100 g of fraction were not included in Fig 4.

^d^ n = 2;—not measured. The values in parentheses indicate the estimated error of the fit of the titration data for ER_50_ (after averaging the experimental data points, typically n = 3 replicates), i.e. great water dock root F1 has an ER_50_ for [CT]/[BSA] of 5.4 (±0.7).

**Fig 5:**
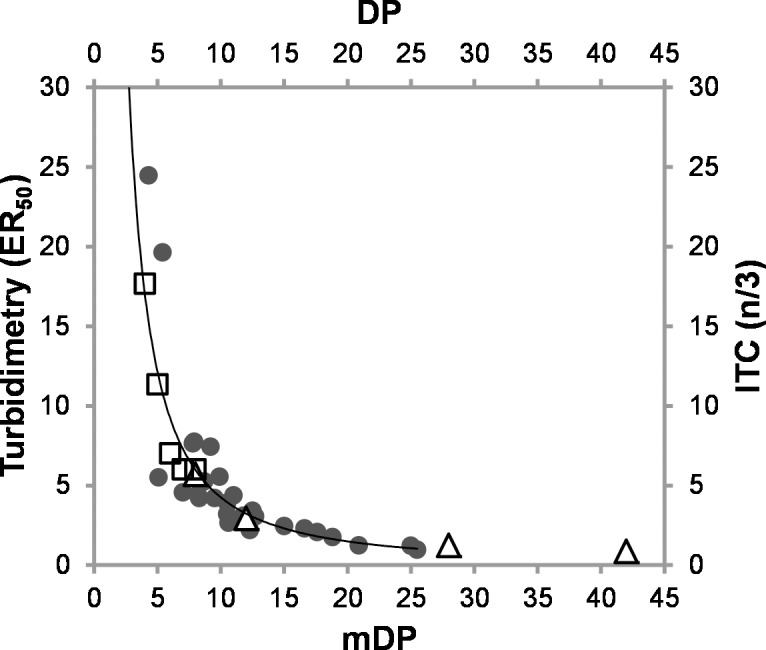
Turbidimetry data from the condensed tannin-BSA study overlaid with published isothermal titration calorimetry (ITC) results: ● –turbidimetry data fitted to a power function, Δ –ref. [19], and ϒ –ref. [22]; *Note*: the stoichiometric number (n) from ITC was divided by 3; ER_50_ –half maximal effective ratio; mDP–mean degree of polymerisation; DP–degree of polymerisation of oligomers.

As shown in Fig 4A and 4B, CT average size is strongly correlated with CT-protein interactions and interestingly the same trend was found in ITC studies, which explored the interactions of B-type CT fractions from sainfoin (at pH 6) [19] and purified PC oligomers from cocoa (at pH 4) [22] with BSA. The stoichiometric ratios from ITC give an excellent overlay with the turbidimetry data when the ITC data are divided by 3 (Fig 5) and show a consistent trend across two different experimental techniques. This might be explained by differences in the experimental method such as fast stirring during ITC experiments that would break up large aggregates and lead to an overall larger surface area. The turbidimetry measurements were carried out in 20 s intervals between additions, whereas the ITC was set up to wait for thermal equilibrium up to 360 s prior to the next addition [19]. Therefore, the CT and protein have more time to bind to each other. It is possible that these variations in the experimental procedure may have been enough to result in the systematic difference in the stoichiometry between the ITC and turbidimetry data.

Only moderate correlations were found when ER_50_ was expressed on a mass basis [(mg/mL)/(mg/mL)] versus mDP or amw (Fig 4E and 4F). By plotting the ER_50_ values of just the B-type CT samples against mDP (*r* = -0.723; *p*<0.01; df = 19) or amw (*r* = -0.716; *p*<0.01; df = 19) a strong correlations were obtained. These results can be explained by the fact that mass-based plots make no allowance for polymer size and this thus also supports the finding that size (average molecular weight) was the most important CT feature across all tannin types whether B-type, A-type or galloylated CT. The other CT characteristics, such as percentages of PC or *cis-*flavan-3-ols within CT, gave no significant correlations whether expressed on a molar (Fig 4C and 4D) or a mass basis (Fig 4G and 4H).

Insoluble CT fractions may have a stronger effect on CT-BSA aggregation. In turbidimetry experiments with BSA and CT there was a significant correlation between ER_50_ and mDP for both, soluble and insoluble samples (>50 g CT/100 g fraction). However this correlation was stronger for insoluble samples (*r* = -0.938 and *r*_s_ = -0.923, *p*<0.01, df = 13) than for soluble samples (*r* = -0.889 and *r*_s_ = -0.917, *p*<0.01, df = 14). This supports recent findings that insoluble PC-salivary protein complexes bind more strongly to oral cells than soluble complexes with PC of lower mDP [42].

### CT-Gelatin Aggregation by Turbidimetry

**Fig 6:**
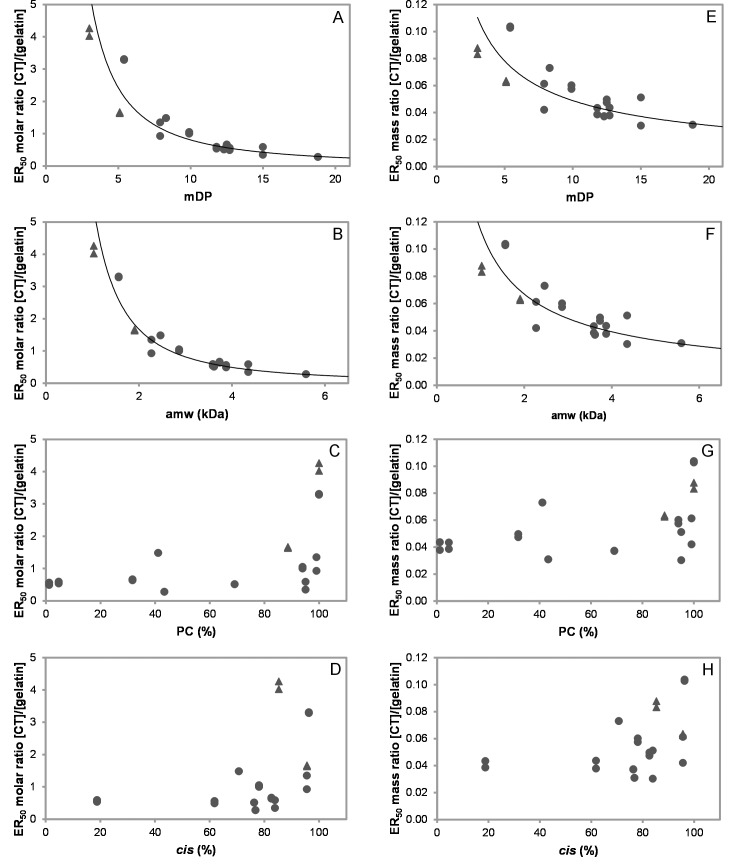
Influence of condensed tannin (CT) characteristics on gelatin aggregation. ER_50_ –half maximal effective ratio, mDP–mean degree of polymerisation, amw–calculated average molecular weight of CT, PC–procyanidins, *cis*–*cis*-flavan-3-ols; ● –B-type CT, ▲ –B-type galloylated CT. Values corrected for CT content; CT fractions of >30 g CT/100 g of fraction; all data points are shown on the graph (for more details see Table 2); (A, B, E, F) fitted to power function for one replicate; (A) ER_50_ [CT]/[gelatin] (M/M) versus mDP, *R*^2^ = 0.92 (*r* = -0.961; *p*<0.01; df = 12 and *r*_s_ = -0.951 *p*<0.01; df = 12); (B) ER_50_ [CT]/[gelatin] (M/M) versus amw (kDa), *R*^2^ = 0.96 (*r* = -0.981; *p*<0.01; df = 12 and *r*_s_ = -0.958; *p*<0.01; df = 12); (C) ER_50_ [CT]/[gelatin] (M/M) versus PC (%); (D) ER_50_ [CT]/[gelatin] (M/M) versus *cis* (%); (E) ER_50_ [CT]/[gelatin] (mg/mL)/(mg/mL) versus mDP, *R*^2^ = 0.77 (*r* = -0.861; *p*<0.01; df = 12 and *r*_s_ = -0.854; *p*<0.01; df = 12); (F) ER_50_ [CT]/[gelatin] (mg/mL)/(mg/mL) versus amw (kDa), *R*^2^ = 0.83 (*r* = -0.897; *p*<0.01; df = 12 and *r*_s_ = -0.879; *p*<0.01; df = 12); (G) ER_50_ [CT]/[gelatin] (mg/mL)/(mg/mL) versus PC (%); (H) ER_50_ [CT]/[gelatin] (mg/mL)/(mg/mL) versus *cis* (%).

Next, a subset of the most representative CT samples was titrated into gelatin solutions. Very significant correlations were also observed between ER_50_ (M/M) versus mDP or amw (Fig 6A and 6B). As seen with BSA, a larger number of small CT molecules were needed to aggregate gelatin compared to large CT molecules. For example, the CT:gelatin ratio was 3.3:1 with cocoa bean F2 (mD = 5.4), 1.1:1 with willow bark F2 (mDP = 9.9) and 0.3:1 with willow bark F3 (mDP = 15); Tables 1 and 2. This also means that much less CT molecules were needed to aggregate gelatin compared to BSA; e.g. the ER_50_ for cocoa was 3 for gelatin, but 20 for BSA.

However, there was one noticeable difference between the BSA and gelatin results. CT-gelatin aggregation also revealed a strong positive correlation on a mass basis between ER_50_ [(mg/mL)/(mg/mL)] versus mDP or amw (Fig 6E and 6F). As before, the molar percentages of PC or *cis*-flavan-3-ols showed almost zero correlations (Fig 6C, 6D, 6G and 6H).

The CT fractions had a stronger tendency to interact with the proline-rich gelatin than with the globular BSA as was observed previously with sorghum PC by ITC [43]. Fig 6A shows that the correlations between ER_50_ (molar basis) and mDP or amw (Fig 6E and 6F) were much stronger during the CT-gelatin complexation, than in the BSA study (mDP: *r* = -0.704; amw: *r* = -0.758; *p*<0.05; df = 12). Therefore, the influence of mDP on the ER_50_ is much higher for the proline-rich gelatin than the globular BSA. This is likely to be due to the higher affinity of CT to the more flexible gelatin. Proline-rich proteins possess randomly coiled structures that offer more binding sites than the globular BSA, which lacks proline on the surface [13].

### CT-BSA Interactions by Circular Dichroism

**Fig 7:**
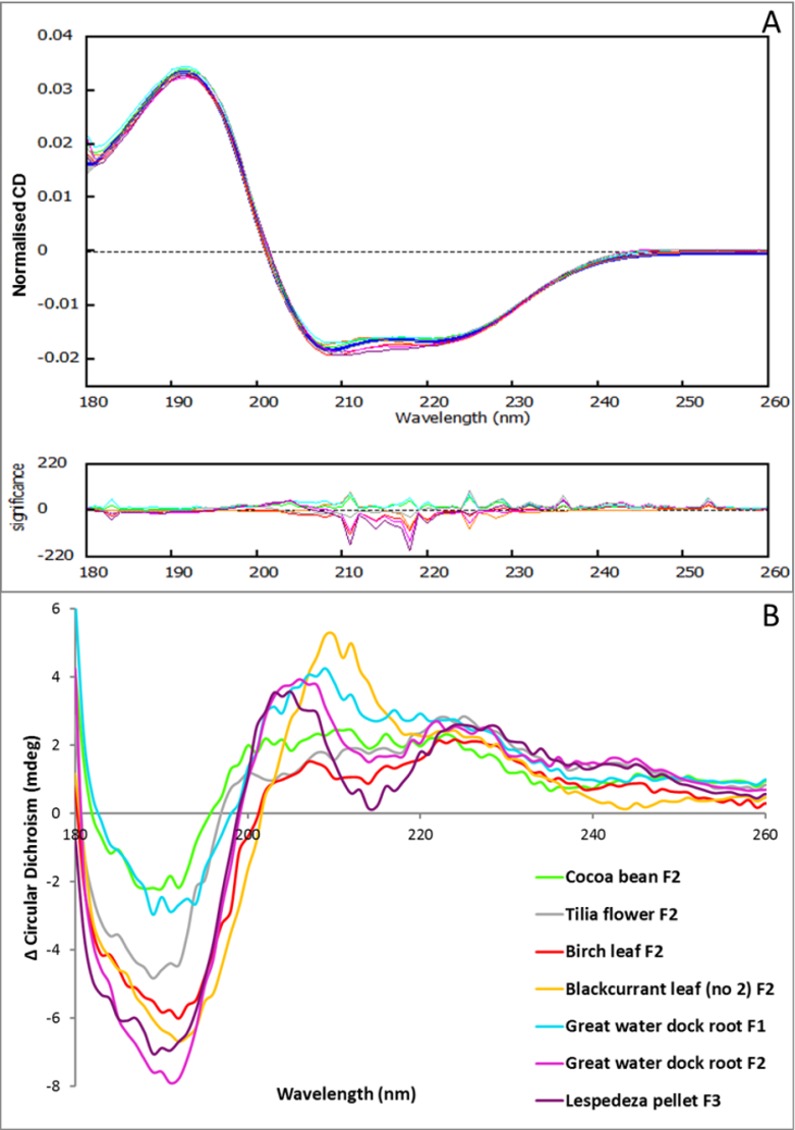
Condensed tannin (CT) interactions study with BSA by circular dichroism (CD). (A) Normalised CD spectra from qBiC software of BSA (reference in dark blue) and of BSA upon interactions with CT fractions (see legend in B); (B) CD difference spectrum of BSA treated with CT fractions calculated by subtracting the ‘CT only’ CD spectra (CD_CT_) and the ‘BSA only’ CD spectra (CD_BSA_) from the spectrum recorded with the CT and BSA mix (CD_BSA_CT_): ΔCD = CD_BSA_CT_ - (CD_CT_ + CD_BSA_).

CD measurements were carried out to investigate how binding of CT to BSA influences its secondary structure. The CD spectra of BSA at pH 6 in the absence of CT showed two negative minima at ~209 nm and ~222 nm and a positive maximum at ~191 nm (Fig 7A), and were similar to reported CD spectra at pH 7.7 [44]. A slight qualitative change in the protein CD spectra was reported during PC oligomer-elastase interactions at pH 7 [17], and for human salivary protein fragment IB7_14_ upon binding to catechin-4α,8-catechin at pH 3.5 in the presence of ethanol [45]. In the current study, slight changes were also observed in the CD spectra after addition of CT fractions, with the most pronounced differences at 190 nm (Fig 7B). Here, we quantified these slight changes (Table 3): the weighted spectral difference mode of the qBiC software weights the comparison of the CD spectra with higher sensitivity at positive or negative CD peaks [46]. Identical spectra would have a weighted spectral difference score (Z-score) of zero. The statistical analysis indicated that all our samples had Z-scores >2 (Table 3), which demonstrated that the CD spectra were clearly different. There were no obvious explanations for the magnitude of these Z-scores in terms of mDP, PC/PD or *cis*-/*trans*-flavan-3-ol ratios. Therefore, although the BSA spectra showed clear differences after adding a range of CT fractions (Fig 7A), the overall amplitude of these differences when measured as weighted spectral differences could not be directly correlated to CT structural characteristics.

**Table 3:** Changes in BSA secondary structure upon binding of condensed tannins (CT).

BSA ± CT fraction	% BSA secondary structure					Weighted spectral difference		Ratio ^a^	
	α-helix	β-sheet	Turn	Unordered	NRMSD ^b^	Similarity	Z-score	[CT]/[BSA] (M/M)	[CT]/[BSA] [(mg/mL)/ (mg/mL)]
BSA	54.6	7.0	11.6	26.8	0.029				
*Tilia* flower F2 + BSA	54.4	7.5	11.5	26.6	0.087	0.00085210	11.9	18.2	0.63
Cocoa bean F2 + BSA	54.0	8.1	11.7	26.2	0.071	0.00089605	12.6	21.7	0.51
Birch leaf F2 + BSA	52.2	8.5	11.8	27.6	0.084	0.00069890	9.2	11.7	0.44
Lespedeza pellet F3 + BSA	49.9	11.7	11.2	27.2	0.079	0.00101430	14.7	4.3	0.49
Blackcurrant leaf (no. 1) F2 + BSA	47.8	12.2	11.9	28.1	0.071	0.00065412	8.4	9.7	0.53
Great water dock root F1 + BSA	50.9	11.3	11.7	26.1	0.086	0.00125030	18.9	15.1	0.23
Great water dock root F2 + BSA	51.6	10.1	11.6	26.8	0.092	0.00108090	15.9	15.9	0.46

^a^ corrected for CT content (g CT/100 g fraction).

^b^ normalised root mean square deviation (i.e. goodness of fit).

The analysis of the CD spectral data shows that the secondary structure of BSA consists of 54.6% (Table 3) α-helix, which is in agreement with results from the literature that were performed at neutral pH with de-fatted BSA [47] (average 53.4%). The addition of CT caused apparent conformational changes of the BSA secondary structure. Blackcurrant leaf (no. 1) F2 generated the largest change in the calculated α-helix content i.e. from 54.6% to 47.8% and this was accompanied by an increase in the β-sheet content from 7.0% to 12.2% (Table 3). It can be seen that the calculated content of α-helices was higher after addition of PC-rich CT fractions (e.g. 54.4% for *Tilia* flower F2) than with PD-rich CT fractions (e.g. 49.9% for lespedeza pellet F3) or with galloylated CT fractions (e.g. 51.6% for great water dock root F2). The apparent α-helix content decreased (*r* = -0.941; *p*<0.01; df = 5) and the apparent β-sheet content increased (*r* = 0.916; *p*<0.05; df = 5) as the molar percentage of PD increased. An increase in β-sheet formation has been observed previously during thermal denaturation of BSA by FTIR, which was linked to intermolecular interaction between protein molecules [48]; it also showed a concomitant loss of α-helix content during unfolding in a surfactant study by CD [44]. These results indicate a link between molecular conformation and aggregation. The CT in concentrations used at measurement conditions in this study do not show a direct effect on the apparent percentage of unordered secondary structure of the globular BSA (Table 3). However, there is some evidence that CT can stabilise collagen matrices [49] and this can be seen even at the molecular level, where a reduction of conformational disorder was calculated for a proline-rich peptide [45].

Both galloylated samples (great water dock leaf F1 and F2) induced a slight loss of α-helix and slight increase in β-sheet contents in BSA, as was also found in the human serum albumin-epigallocatechin gallate complex, where slight changes were observed to the secondary structure, i.e. α-helix content decreased from 57 to 54% at a 1:3 molar ratio [50]. In agreement with the literature, where PC size did not change the secondary structure of globular elastase [17], mDP had no effect on BSA conformation (data not shown), Table 3 also shows that the changes in the α-helix and β-sheet contents were not correlated with CT concentrations and this agrees with the literature, where similar molar ligand ratios had been used for BSA-flavonol binding in the presence of ethanol [51].

### CT-BSA Interactions by Tryptophan Fluorescence Quenching

Measurements of fluorescence quenching have been used to explore tannin-protein interactions [52]. The fluorescence of BSA has been attributed to Trp 134 in the surface region of subdomain IB and to Trp 213 in the hydrophobic binding pocket, the Suldow I site in subdomain IIA [53] due to the indole group of tryptophan that absorbs at ~280 nm and emits at ~340 nm. Since it was reported that PC trimers do not exhibit fluorescence [54], all 35 CT fractions were first screened for any fluorescence and then only the 9 non-fluorescent CT samples were used.

Stern-Volmer plots, *F*_0_/*F* versus [CT], gave a concave deviation towards the y-axis, which is generally interpreted as the presence of dynamic and static quenching [37]. The use of the initial linear part of the graph resulted in Stern-Volmer plots of good linear fits (e.g. *F*_0_/*F* versus [CT] (M), *R*^2^ = 0.99–1.00, S2 Fig). In general, diffusion-controlled quenching in aqueous solution has an apparent bimolecular quenching constant (*k*_q_^app^) of ≤10^10^ M^-1^ s^-1^ for tryptophan [37]. In the present study, blackcurrant leaf F2 (no. 2) showed the lowest quenching ability, *K*_SV_ = 0.3×10^5^ M^−1^ (Table 4) and gave a calculated *k*_q_^app^ of 6×10^12^ M^-1^ s^-1^, which indicates a static quenching. This is in line with the literature on epicatechin-BSA interactions [18].

**Table 4:** Estimated quenching parameters, Stern-Volmer constant (*K*_SV_), for the interactions of condensed tannins (CT) with BSA.

CT fraction	*K*_SV_		n
	(M^−1^)	[(mg/mL)^−1^]	
Great water dock root F1 ^a^	1.3×10^5^ (±6.0×10^3^)	224.2 (±21.3)	3
Great water dock root F2	2.4×10^5^ (±3.9×10^3^)	127.2 (±2.1)	3
Great water dock root F3 ^a^	4.5×10^5^ & 4.5×10^5^	90.6 & 91.4	2
Birch leaf F2	1.8×10^5^ (±5.7×10^3^)	73.1 (±2.3)	3
Birch leaf F3	3.9×10^5^ & 3.7×10^5^	73.9 & 70.1	2
Blackcurrant leaf (no. 2) F2	0.3×10^5^ (±0.5×10^3^)	12.2 (±0.2)	3
Blackcurrant leaf (no. 2) F3	1.4×10^5^ (±13.0×10^3^)	26.9 (±2.5)	3
Pine bud F2	0.9×10^5^ (±1.7×10^3^)	27.3 (±0.5)	3
Pine bud F3	2.1×10^5^	39.5	1

^a^ <50 g CT/100 g fraction; n–number of replicates (the average presented for n = 3, standard deviation in parentheses).

**Fig 8:**
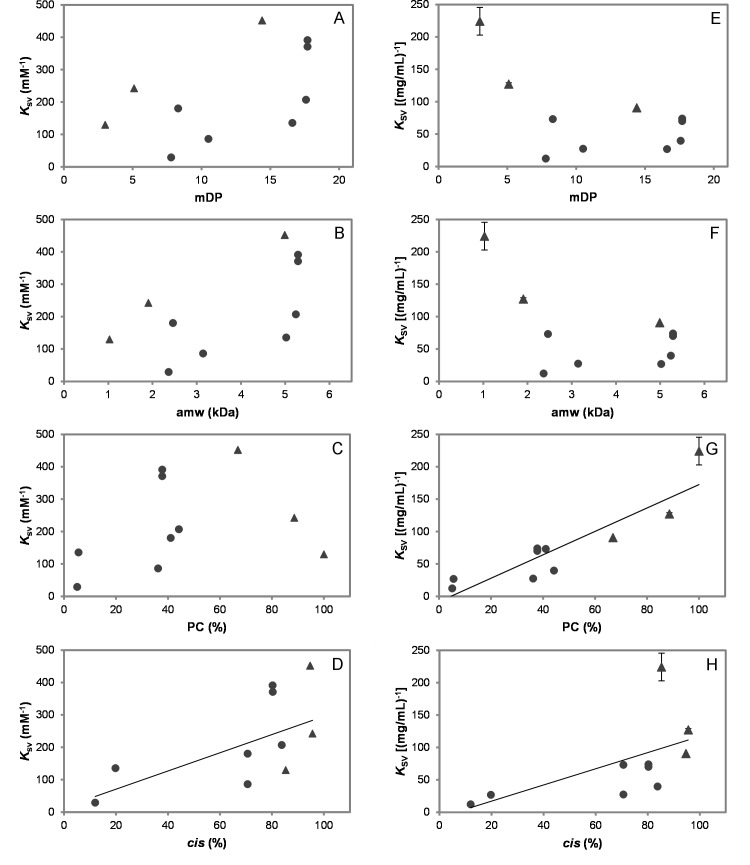
Fluorescence quenching of tryptophan in BSA in relation to condensed tannin (CT) characteristics. *K*_SV_−Stern-Volmer quenching constant, i.e. slope obtained from linear part of Stern-Volmer plot fitted to linear regression: *F*_0_/*F* versus [CT]; mDP–mean degree of polymerisation; amw–average molecular weight of CT; PC–procyanidins, *cis*–*cis*-flavan-3-ols; ● –B-type CT, ▲ –B-type galloylated CT. Values corrected for CT content, error bars indicate the standard deviation of n = 3 replicates (if n<3, all data points are shown; for more details see Table 4); (A) *K*_SV_ (mM^-1^) versus mDP; (B) *K*_SV_ (mM^-1^) versus amw (kDa); (C) *K*_SV_ (mM^-1^) versus PC (%); (D) *K*_SV_ (mM^-1^) versus *cis* (%), *R*^2^ = 0.39, (*r*_s_ = 0.678; *p*<0.05; df = 9); (E) *K*_SV_ [(mg/mL)^-1^] versus mDP; (F) *K*_SV_ [(mg/mL)^-1^] versus amw (kDa); (G) *K*_SV_ [(mg/mL)^-1^] versus PC (%), *R*^2^ = 0.81 (*r* = 0.899; *p*<0.01; df = 9); (H) *K*_SV_ [(mg/mL)^-1^] versus *cis* (%), *R*^2^ = 0.34, (*r*_s_ = 0.887; *p*<0.01; df = 9).

The *K*_SV_ values (Table 4) were then plotted against the different CT characteristics (Table 1). There was a very significant strong correlation between *K*_SV_ values and PC contents when the *K*_SV_ calculations were based on mass concentration [55] (Fig 8G); however, this correlation was not observed when expressed on a molar basis (Fig 8C). However, the three PC-rich CT were also the only three samples that contained galloyl groups and it is, therefore, possible that these galloyl groups may have contributed to this strong correlation between *K*_SV_ values and PC contents. In fact, another study also found that galloyl groups enhanced the binding affinity of PC to human salivary α-amylase as determined by fluorescence quenching [56]. However, a closer look revealed that the PC-rich sample with the highest percentage of galloylation (54%, great water dock root F2) did not have the highest affinity to BSA, i.e. *K*_SV_ = 127.2 (mg/mL)^−1^; the highest affinity was instead observed with great water dock root F1, which had 34% of galloylation and gave *K*_SV_ = 224.2 (mg/mL)^−1^. This suggested that the presence of PC rather than galloyl groups played a crucial role in the affinity towards the subdomain IB and subdomain IIA of BSA.

There was a strong, significant correlation between *K*_SV_ expressed on a mass basis and *cis*-flavan-3-ol content (Fig 8H). *K*_SV_ expressed on a molar basis and *cis*-flavan-3-ol content were moderately correlated (Fig 8D). No correlations could be found between CT average size and *K*_SV_ (Fig 8A, 8B, 8E and 8F). It is interesting that these CT yielded *K*_SV_ values (0.3 to 4.5×10^5^ M^−1^; Table 4) that were similar to values obtained for pure ellagitannins [52] (0.4 to 3.1×10^5^ M^−1^) with BSA in the same buffer. Although, ellagitannin dimers had higher *K*_SV_ values than monomers [52], no clear size effect could be detected here with this CT panel (Fig 8).

A bathochromic shift of the tryptophan fluorescence was observed with the galloylated CT; for example, the great water dock root F2 sample contributed to a shift of λ_em max_ from ~350 nm to ~380 nm. A red shift has also been observed for epigallocatechin gallate-human serum albumin interactions [50]. This shift indicated that the surrounding environment of tryptophan became more polar, possibly due to the unfolding of BSA [32]. No bathochromic shift was observed for non-galloylated samples (data not shown).

## Conclusions

A large panel of CT with different structural features was isolated from a diverse set of plants in order to cover a wide range of average oligomer/polymer sizes and structures. The mean degree of polymerisation and average molecular weight correlated very significantly to the efficacy of CT to aggregate BSA and gelatin in a turbidimetry study. The average size of the CT, rather than the hydroxylation pattern or stereochemistry of the flavan-3-ol subunits, was most important for aggregation. The data can be approximated with two linear fits, which intersected at an mDP of ~7 or ~2000 Da. This means that the smallest CT with the relatively largest effect on protein aggregation would have an mDP of ~7. A similar trend was observed in a procyanidin-BSA study by ITC where a CT hexamer of 1721 Da had an optimal binding stoichiometry [22]. Interestingly, the greatest effect on bioactivity was also observed at around ~2000 Da in two unrelated studies. Immunological effects of CT showed that mDP of 6.5 and 9.1 had the greatest effect on the activation of porcine γδ T-cells compared to CT of lower mDP values [57]. Similarly, the highest inhibition of Hepatitis C virus RNA expression was observed with mDP 7.7 in tests that had explored mDP values from ~1 to 14 [58]. It remains to be seen what the mechanisms are behind this tannin threshold and whether this applies more widely across different biological systems. This type of information may also prove helpful for breeding new plant varieties with highly active CT as nutraceuticals [59].

The molar percentage of procyanidins and *cis*-flavan-3-ols was positively correlated to the Stern-Volmer quenching constant that was obtained from tryptophan fluorescence quenching. However, pyrogallol groups of prodelphinidins (i.e. B-ring of flavan-3-ol subunits) or esterified galloyl groups appeared to slightly change the apparent α-helix and β-sheet contents in a circular dichroism study. Taken together, these results indicated that the interaction between CT and BSA was most sensitive to the presence of procyanidins, whereas the secondary structure of BSA was most influenced by the presence of prodelphinidins. Protein aggregation was solely affected by CT average size.

## Supporting Information

S1 Fig Lack of observed correlation between condensed tannin (CT) characteristics among all CT. mDP–mean degree of polymerisation, PD–prodelphinidin, *cis*–*cis*-flavan-3-ols; ● –B-type CT, ▲ –B-type galloylated CT, ■ –B-type with A-type linkages; (A) PD (%) versus mDP, (*r*_s_ = 0.434; *p*<0.01; df = 35); (B) *cis* (%) versus mDP; (C) *cis* (%) versus PD (%),(*r*_s_ = -0.619; *p*<0.01; df = 35).
(PDF)

S2 Fig Fluorescence quenching of tryptophan in BSA by condensed tannins (CT), as Stern-Volmer plots. Error bars indicate standard deviation of repeats; data corrected for CT content; (A) titration points of aliquots with increasing CT concentration (M); (B) titration points of aliquots with increasing CT concentration (M) at linear part of the plot (up to *F*_0_/*F*≈2.2) fitted to linear regression, all *R*^2^ = 0.99–1.00; (C) titration points of aliquots with increasing CT concentration (mg/mL); (D) titration points at linear part of the plot (up to *F*_0_/*F*≈2.2) fitted to linear regression, all *R*^2^ = 0.99–1.00.
(PDF)

S1 Table Composition of condensed tannins in terms of flavan-3-ol terminal and extension units (as molar percentages). *Note*: A few of these fractions were reported previously [4, 9] and are included for clarity purposes.
(PDF)
