# Supplementary material for: Identification of Structural Features of Condensed Tannins That Affect Protein Aggregation
Source: PLoS One. 2017 Jan 26;12(1):e0170768. doi: 10.1371/journal.pone.0170768 (PMC5268436; doi:10.1371/journal.pone.0170768)
Supplement: S1 Fig — mDP–mean degree of polymerisation, PD–prodelphinidin, cis–cis-flavan-3-ols; ● –B-type CT, ▲ –B-type galloylated CT, ■ –B-type with A-type linkages; (A) PD (%) versus mDP, (rs = 0.434; p<0.01; df = 35); (B) cis (%) versus mDP; (C) cis (%) versus PD (%),(rs = -0.619; p<0.01; df = 35). (PDF) [file pone.0170768.s001.pdf]

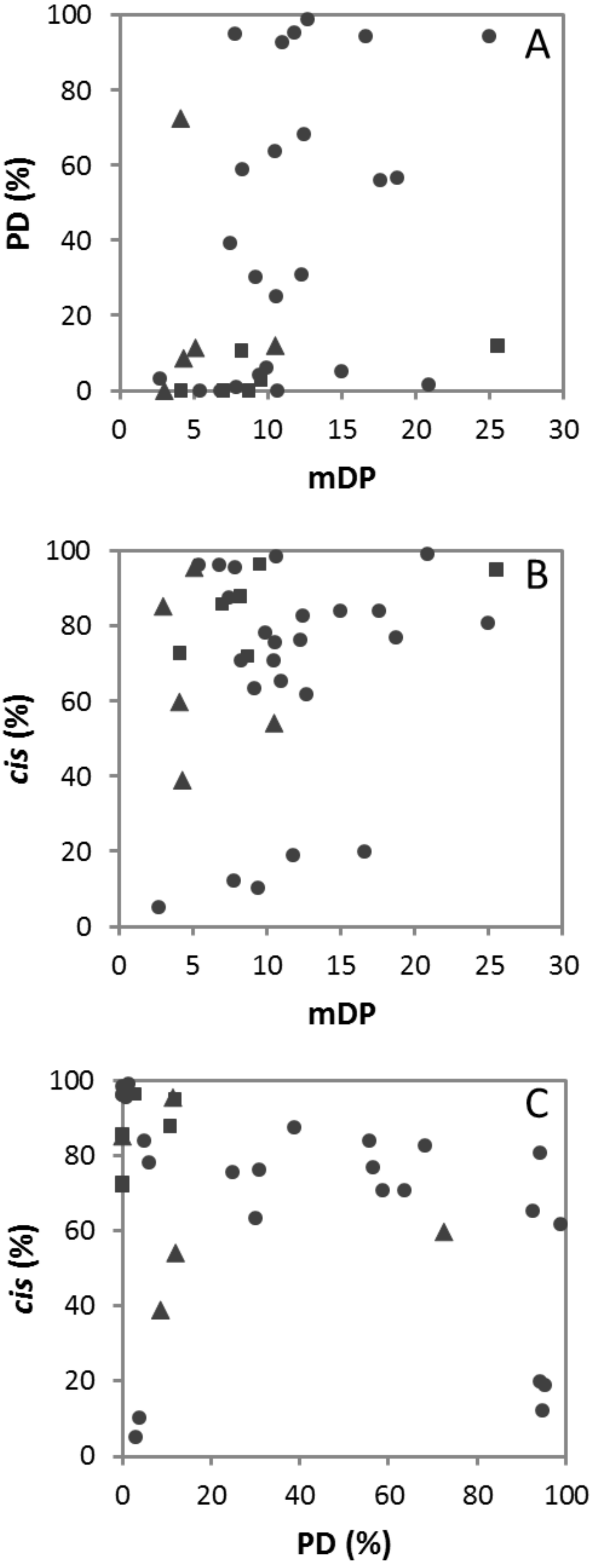

S1 Fig. Lack of observed correlation between condensed tannin (CT) characteristics among all CT. mDP – mean degree of polymerisation, PD – prodelphinidin, *cis* – *cis*-flavan-3-ols; ● – B-type CT, ▲ – B-type galloylated CT, ■ – B-type with A-type linkages; values corrected for CT content; (A) PD (%) versus mDP, (r_s=0.434; p<0.01; df=35); (B) *cis* (%) versus mDP; (C) *cis* (%) versus PD (%),(r_s=-0.619; p<0.01; df=35).
