# Supplementary material for: Identification of Structural Features of Condensed Tannins That Affect Protein Aggregation
Source: PLoS One. 2017 Jan 26;12(1):e0170768. doi: 10.1371/journal.pone.0170768 (PMC5268436; doi:10.1371/journal.pone.0170768)
Supplement: S2 Fig — Error bars indicate standard deviation of repeats; data corrected for CT content; (A) titration points of aliquots with increasing CT concentration (M); (B) titration points of aliquots with increasing CT concentration (M) at linear part of the plot (up to F0/F≈2.2) fitted to linear regression, all R2 = 0.99–1.00; (C) titration points of aliquots with increasing CT concentration (mg/mL); (D) titration points at linear part of the plot (up to F0/F≈2.2) fitted to linear regression, all R2 = 0.99–1.00. (PDF) [file pone.0170768.s002.pdf]

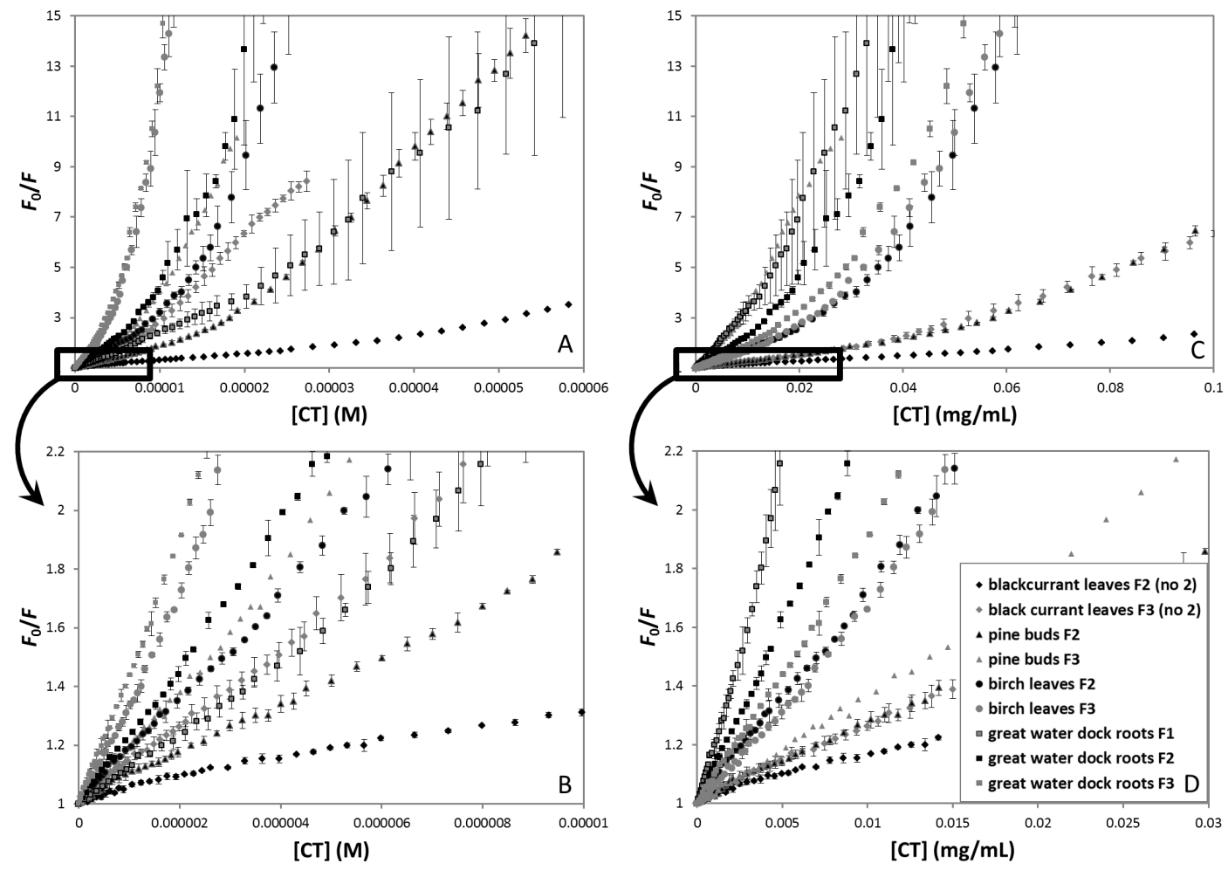

S2 Fig. Fluorescence quenching of tryptophan in BSA by condensed tannins (CT), as Stern-Volmer plots. Values corrected for CT content; error bars indicate standard deviation of repeats; (A) titration points of aliquots with increasing CT concentration (M); (B) titration points of aliquots with increasing CT concentration (M) at linear part of the plot (up to F_0/F≈2.2) fitted to linear regression, all R^2=0.99-1.00; (C) titration points of aliquots with increasing CT concentration (mg/mL); (D) titration points at linear part of the plot (up to F_0/F≈2.2) fitted to linear regression, all R^2=0.99-1.00.
