# Supplementary material for: Identification of Structural Features of Condensed Tannins That Affect Protein Aggregation
Source: PLoS One. 2017 Jan 26;12(1):e0170768. doi: 10.1371/journal.pone.0170768 (PMC5268436; doi:10.1371/journal.pone.0170768)
Supplement: S1 Table — Note: A few of these fractions were reported previously [4, 9] and are included for clarity purposes. (PDF) [file pone.0170768.s003.pdf]

S1 Table. Composition of condensed tannins in terms of flavan-3-ol terminal and extension units (as molar percentages).

Note: A few of these fractions were reported previously [4, 9] and are included for clarity purposes.

CT fraction	Tannin subunit composition (%)													
	GC	EGC	C	EC	GC -BM	EGC -BM	C -BM	EC -BM	ECg	ECg -BM	GCg	GCg -BM	EGCg	EGCg -BM
Great water dock root F1 [a]	0.0	0.0	12.6	5.7	0.0	0.0	2.1	44.9	14.9	19.8	nd	nd	nd	nd
±	0.0	0.0	0.2	0.2	0.0	0.0	0.2	0.1	0.1	0.1				
Great water dock root F1 [b]	0.0	0.0	27.8	8.7	0.0	0.0	1.3	30.0	19.0	13.2	nd	nd	nd	nd
±	0.0	0.0	0.1	0.1	0.0	0.0	0.2	0.1	0.1	0.1				
Great water dock root F2	0.0	0.0	4.1	0.8	*	5.2	0.3	35.2	14.7	39.7	nd	nd	nd	nd
±	0.0	0.0	0.1	0.1	0.0	0.1	0.3	0.1	0.1	0.1				
Great water dock root F3	0.0	0.0	2.5	0.8	2.3	19.6	0.6	40.3	3.7	30.4	nd	nd	nd	nd
±	0.0	0.0	0.0	0.1	0.3	0.1	0.4	0.1	0.0	0.1				
Hazelnut pericarp F1 [a]	1.0	0.0	18.0	1.8	5.5	2.3	32.7	34.2	0.6	1.0	nd	nd	nd	nd
±	0.1	0.0	0.0	0.0	0.1	0.2	0.1	0.1	0.1	0.1				
Hazelnut pericarp F1 [b]	0.9	0.0	19.8	1.8	5.3	2.2	35.0	33.4	0.7	0.9	nd	nd	nd	nd
±	0.1	0.0	0.0	0.0	0.1	0.2	0.1	0.1	0.1	0.1				
Hazelnut pericarp F2	0.0	0.0	7.9	0.8	6.0	5.6	32.0	44.1	0.9	2.8	nd	nd	nd	nd
±	0.0	0.0	0.1	0.1	0.2	0.1	0.1	0.1	0.1	0.2				
Shea meal F2 [4]	4.1	2.5	1.1	0.0	20.7	9.4	3.5	12.6	12.6	6.6	3.8	6.1	9.8	16.0
±	0.1	0.1	0.0	0.0	0.2	0.3	0.1	0.0	0.0	0.1	0.0	0.0	0.1	0.1
									**PC A-type trimer I**	**PC A-type trimer II**	**PC A-type dimer I**	**PC A-type dimer II**	**PC A-type trimer -BM**	**PC A-type dimer -BM**
Bilberry leaf F2	0.0	1.0	1.3	8.5	0.7	0.8	1.2	81.0	0.7	0.0	0.0	0.0	2.3	2.5
±	0.0	0.1	0.0	0.1	0.2	0.1	0.6	0.0	0.1	0.0	0.0	0.0	0.1	0.0
Blackthorn flower F2	0.0	0.0	9.8	2.3	0.0	0.0	12.2	56.5	0.0	0.0	15.5	1.8	0.6	1.3
±	0.0	0.0	0.1	0.1	0.0	0.0	0.2	0.1	0.0	0.0	0.1	0.2	0.1	0.2
Cinnamon bark F2 [9]	0.0	0.0	6.8	3.1	0.0	0.0	4.6	66.6	5.7	1.2	1.8	0.0	4.6	5.5
±	0.0	0.0	0.1	0.1	0.0	0.0	0.2	1.1	0.2	6.6	0.1	0.0	0.1	0.1
Cowberry leaf F2	0.0	0.0	8.8	0.7	0.0	0.0	13.8	57.6	0.8	*	4.1	0.3	7.2	6.8
±	0.0	0.0	0.0	0.0	0.0	0.0	0.1	0.0	0.0	0.0	0.0	0.1	0.0	0.0
Heather flower F2	0.0	0.0	7.9	3.0	0.0	9.9	3.2	68.7	2.6	0.8	*	*	*	3.9

±	0.0	0.0	0.0	0.0	0.0	0.1	0.0	0.0	0.0	0.1	0.0	0.0	0.0	0.0
Heather flower F3	0.0	0.0	2.7	1.0	0.0	11.8	2.2	81.1	0.4	0.2	0.0	0.0	0.0	0.8
±	0.0	0.0	0.0	0.0	0.0	0.1	0.1	0.0	0.0	0.0	0.0	0.0	0.0	0.0
Birch leaf F2	4.4	*	7.7	*	11.0	43.6	6.3	27.1	nd	nd	nd	nd	nd	nd
±	0.1	0.0	0.1	0.0	0.1	0.1	0.1	0.1						
Birch leaf F3	2.0	0.0	3.5	0.2	9.4	50.9	5.0	29.1	nd	nd	nd	nd	nd	nd
±	0.1	0.0	0.1	0.4	0.1	0.0	0.0	0.0						
Blackcurrant leaf (no 1) F2	6.5	0.9	1.0	0.1	71.4	16.5	2.3	1.3	nd	nd	nd	nd	nd	nd
±	0.1	0.1	0.1	0.2	0.1	0.1	0.2	0.0						
Blackcurrant leaf (no. 2) F2	8.8	2.1	1.7	0.2	75.3	8.8	2.3	0.9	nd	nd	nd	nd	nd	nd
±	0.1	0.1	0.0	0.1	0.1	0.1	0.1	0.0						
Blackcurrant leaf (no. 2) F3	4.1	0.8	1.0	0.1	72.9	16.6	2.2	2.3	nd	nd	nd	nd	nd	nd
±	0.1	0.1	0.1	0.1	0.1	0.1	0.2	0.1						
Cider apple beverage F2	0.7	0.0	3.4	9.2	7.6	30.6	0.7	47.8	nd	nd	nd	nd	nd	nd
±	0.1	0.0	0.1	0.1	0.1	0.2	0.2	0.1						
Cider apple powder F2	0.0	0.0	3.7	11.1	0.0	0.0	0.0	85.2	nd	nd	nd	nd	nd	nd
±	0.0	0.0	0.0	0.1	0.0	0.0	0.0	0.1						
Cocoa bean F2	0.0	0.0	2.5	15.9	0.0	0.0	1.2	80.4	nd	nd	nd	nd	nd	nd
±	0.0	0.0	0.1	0.3	0.0	0.0	0.1	0.4						
Hawthorn flower F2	0.0	0.0	0.9	8.4	0.0	0.0	0.5	90.1	nd	nd	nd	nd	nd	nd
±	0.0	0.0	0.0	0.0	0.0	0.0	0.3	0.0						
Hop strobile F2	0.7	*	7.5	1.2	3.8	20.5	12.4	53.8	nd	nd	nd	nd	nd	nd
±	0.1	0.0	0.1	0.1	0.1	0.1	0.1	0.0						
Lespedeza pellet F3	2.7	1.1	0.2	0.1	15.3	75.4	1.1	4.3	nd	nd	nd	nd	nd	nd
±	0.1	0.0	0.0	0.0	0.1	0.1	0.1	0.1						
Pine bud F2	3.6	0.4	5.1	0.4	10.7	49.2	10.0	20.7	nd	nd	nd	nd	nd	nd
±	0.1	0.1	0.1	0.2	0.1	0.1	0.1	0.1						
Pine bud F3	1.7	0.0	3.2	0.8	4.0	50.1	7.3	33.0	nd	nd	nd	nd	nd	nd
±	0.2	0.0	0.1	0.1	0.4	0.1	0.1	0.1						
Redcurrant leaf F2	6.1	0.6	2.0	0.3	24.8	60.9	1.8	3.5	nd	nd	nd	nd	nd	nd
±	0.1	0.1	0.1	0.1	0.1	0.1	0.2	0.1						
Sainfoin aerial part F2	2.1	1.6	2.4	1.9	10.4	54.2	2.5	24.9	nd	nd	nd	nd	nd	nd
±	0.1	0.1	0.1	0.1	0.1	0.1	0.1	0.1						
Tilia flower F2	*	*	2.3	10.4	*	0.9	2.1	84.3	nd	nd	nd	nd	nd	nd
±	0.0	0.0	0.1	0.1	0.0	0.2	0.3	0.1						
Tilia flower F3	0.0	0.0	1.0	3.8	0.0	1.4	*	93.8	nd	nd	nd	nd	nd	nd
±	0.0	0.0	0.2	0.2	0.0	0.3	0.0	0.2						
Walnut leaf F2	0.7	0.0	6.7	0.6	6.3	23.9	10.0	51.7	nd	nd	nd	nd	nd	nd

±	0.3	0.0	0.1	0.1	0.1	0.0	0.1	0.1						
Walnut leaf F3	1.2	0.5	3.3	0.4	13.0	41.9	5.7	34.0	nd	nd	nd	nd	nd	nd
±	0.1	0.2	0.1	0.1	0.1	0.1	0.3	0.1						
White clover flower F2	6.1	1.8	*	0.0	31.7	59.2	0.3	0.8	nd	nd	nd	nd	nd	nd
±	0.0	0.0	0.0	0.0	0.1	0.0	0.0	0.0						
Willow bark F2	*	0.0	9.7	0.4	0.9	5.1	11.3	72.6	nd	nd	nd	nd	nd	nd
±	0.0	0.0	0.0	0.1	0.1	0.1	0.0	0.1						
Willow bark F3	0.0	0.0	6.3	0.3	0.7	4.2	9.1	79.4	nd	nd	nd	nd	nd	nd
±	0.0	0.0	0.1	0.1	0.1	0.2	0.1	0.1						
Yellow iris leaf F2	0.0	0.0	10.3	0.5	0.8	29.4	25.6	33.4	nd	nd	nd	nd	nd	nd
±	0.0	0.0	0.1	0.1	0.0	0.1	0.1	0.1						

[a] corrected for free flavan-3-ols; [b] not corrected for free flavan-3-ols (for comparison purposes only); ± standard deviation; * trace amounts detected; [nd] not detected; CT – condensed tannin; GC – gallocatechin, EGC – epigallocatechin, C – catechin, EC – epicatechin, GC-BM – gallocatechin benzyl mercaptan adduct, EGC-BM – epigallocatechin benzyl mercaptan adduct, C-BM – catechin benzyl mercaptan adduct, EC-BM – epicatechin benzyl mercaptan adduct, ECg – epicatechin gallate, ECg-BM – epicatechin gallate benzyl mercaptan adduct, GCg – gallocatechin gallate, GCg – gallocatechin gallate benzyl mercaptan adduct, PC – procyanidins.
